# Protein phosphatase 2A regulates central sensitization in the spinal cord of rats following intradermal injection of capsaicin

**DOI:** 10.1186/1744-8069-2-9

**Published:** 2006-03-20

**Authors:** Xuan Zhang, Jing Wu, Yongzhong Lei, Li Fang, William D Willis

**Affiliations:** 1 Department of Neuroscience and Cell Biology, The University of Texas Medical Branch, 301 University Blvd., Galveston, TX 77555-1069, USA; 2 Division of Neurosurgery, Department of Surgery, The University of Texas Medical Branch, 301 University Blvd., Galveston, TX 77555-1069, USA

## Abstract

**Background:**

Intradermal injection of capsaicin into the hind paw of rats induces spinal cord central sensititzation, a process in which the responsiveness of central nociceptive neurons is amplified. In central sensitization, many signal transduction pathways composed of several cascades of intracellular enzymes are involved. As the phosphorylation state of neuronal proteins is strictly controlled and balanced by the opposing activities of protein kinases and phosphatases, the involvement of phosphatases in these events needs to be investigated. This study is designed to determine the influence of serine/threonine protein phosphatase type 2A (PP2A) on the central nociceptive amplification process, which is induced by intradermal injection of capsaicin in rats.

**Results:**

In experiment 1, the expression of PP2A protein in rat spinal cord at different time points following capsaicin or vehicle injection was examined using the Western blot method. In experiment 2, an inhibitor of PP2A (okadaic acid, 20 nM or fostriecin, 30 nM) was injected into the subarachnoid space of the spinal cord, and the spontaneous exploratory activity of the rats before and after capsaicin injection was recorded with an automated photobeam activity system. The results showed that PP2A protein expression in the spinal cord was significantly upregulated following intradermal injection of capsaicin in rats. Capsaicin injection caused a significant decrease in exploratory activity of the rats. Thirty minutes after the injection, this decrease in activity had partly recovered. Infusion of a phosphatase inhibitor into the spinal cord intrathecal space enhanced the central sensitization induced by capsaicin by making the decrease in movement last longer.

**Conclusion:**

These findings indicate that PP2A plays an important role in the cellular mechanisms of spinal cord central sensitization induced by intradermal injection of capsaicin in rats, which may have implications in clinical pain therapy.

## Introduction

The phosphorylation and dephosphorylation of proteins are reversible processes, catalyzed by opposing protein kinases and phosphatases (PP), respectively. Such reactions seem to modulate the function of several proteins involved in synaptic transmission, including voltage-gated and ligand-gated channels, ionotropic and metabotropic neurotransmitter receptors, proteins involved in neurotransmitter release and transport, and cytoskeletal proteins [1-8]. These proteins play an important role in the control of many intracellular events. Serine/threonine protein phosphatase, which dephosphorylates serine and threonine protein residues, can be divided into PP1, PP2A, PP2B, PP2C, PP4, PP5, PP6 and PP7 [2]. Among them, PP2A is the most abundant phosphatase in mammalian cells and is expressed at high levels in the central nervous system [1,6]. PP2A regulates many fundamental cellular processes, such as cell division, signal transduction, gene expression, development, the cell cycle, exocytosis, and apoptosis [2,6,9-12]. The function of this protein phosphatase can be inhibited by several drugs, such as okadaic acid (OA), which is a cell permeable molecule that inhibits PP2A *in vitro *at much lower concentrations than PP1 (1:100) [13,14]. Fostriecin is another cell permeable protein phosphatase inhibitor. Since this molecule is on the order of 10^4^–10^5 ^times more selective for PP2A than PP1, fostriecin is the most selective inhibitor known for any member of this class of phosphatases [13].

Strong noxious stimulation of peripheral tissues, such as intradermal injection of capsaicin into the hind paw of rats, induces spinal cord central sensititzation, a process in which an amplified responsiveness of central nociceptive neurons is found. It has been verified that signal transduction pathways involved in central sensitization are composed of several cascades of enzymes that activate intracellular protein kinases. As the phosphorylation state of neuronal proteins is strictly controlled and balanced by the opposing activities of protein kinases and phosphatases, the involvement of phosphatases in these events needs to be investigated.

In the present study, we evaluated the effects of noxious stimulation of peripheral tissues on the expression of PP2A protein in the spinal cord of rats. We also tested the effects of two inhibitors of PP2A, okadaic acid and fostriecin, on the induction and maintenance of central sensitization induced by capsaicin injection, using the spontaneous exploratory activity test. The inhibitors were administered through an intrathecal catheter to detect the role of the spinal cord in changes in these behaviors.

## Results

### Increased expression of PP2A in spinal cord after capsaicin injection

**Figure 1:**
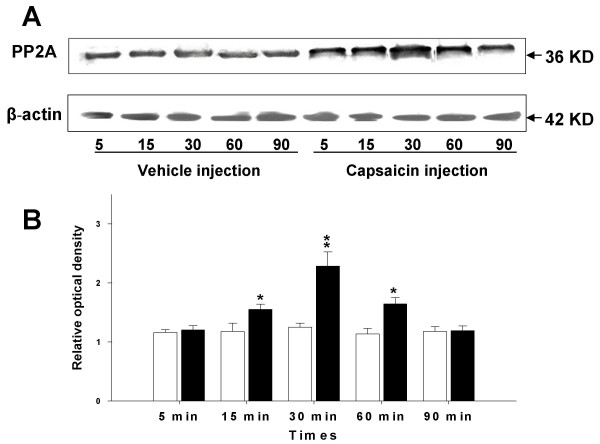
Expression of PP2A protein in the spinal cord at 5, 15, 30, 60, and 90 minutes following vehicle or capsaicin injection in the periphery. (A) Immunoreactive bands of rat spinal cord tissue (L3–L6) against PP2A antibody at 5, 15, 30, 60, and 90 minutes after capsaicin or vehicle injection; the lower row shows the bands produced by the antibody to β-actin as a loading control at the same times. (B) Bar graph summarizing the relative density of the immunoblots of rat spinal cord tissue (L3–L6) with the PP2A antibody to that of β-actin at 5, 15, 30, 60, and 90 minutes after capsaicin or vehicle injection. Open bar: vehicle injection, filled bar: capsaicin injection. N = 6 in each group; the relative densities are expressed as mean ± SEM. *p < 0.05.

To determine if the expression of PP2A protein in the lumber spinal cord of rats changes following noxious stimulation with intradermal injection of capsaicin, a Western blot analysis was performed. The Western blots in Fig. 1A show that the expression of PP2A in the lumbar segments (L_3_-L_6_) of the spinal cord tissue was detected at around 36 kDa. The relative density of immunoblots of PP2A protein from rat spinal cord tissue after intradermal vehicle or capsaicin injection is summarized in Fig 1B. Statistical analysis of these data showed that intradermal injection of capsaicin causes a significant increase in expression of PP2A protein in a time-dependent manner compared with the vehicle injection group. The PP2A protein started to increase in spinal cord tissue by 15 min, peaked at 30 min, and the increase continued for more than 60 min following intradermal injection of capsaicin. Vehicle injection did not result in an obvious increase in the expression of PP2A protein in the lumbosacral spinal cord. In addition, the expression of β-actin did not show any significant change in any of the groups.

### The effect of intrathecal catheter implantation on exploratory behavior

**Figure 2:**
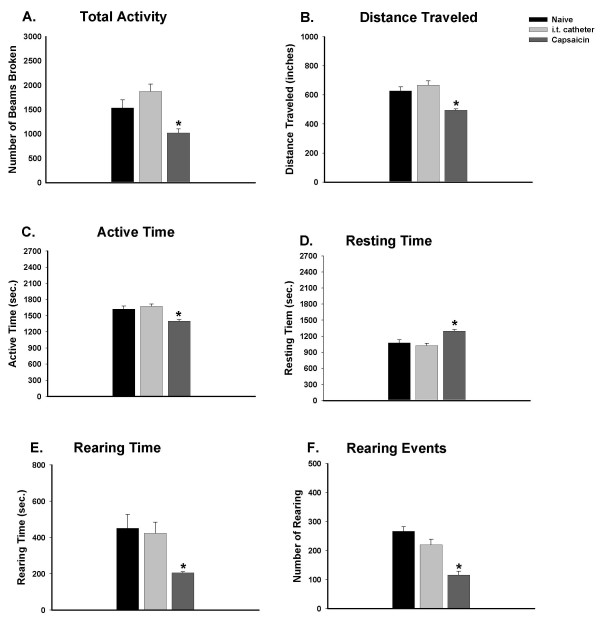
Exploratory activity during a consecutive period of 45 minutes (9 intervals, 5 min/interval) after introduction of the rat into the novel chamber. Compared to naïve rats (n = 14), intrathecal catheter implanted rats (n = 14) do not have significant alterations in exploratory activity. Rats with intradermal injection of capsaicin (1%, 50 μl) (n = 6) demonstrated a reduction of exploratory activity, including decreased number of photobeams broken (A), shortened travel distance (B), decreased active time (C), increased resting time (D), shortened rearing time (E), and decreased number of rearing events (F). The capsaicin-injected group was compared to the other two groups. *p < 0.05.

The spontaneous activity of rats and all six behavioral parameters were examined both before and 5 days after intrathecal implantation of a catheter. Fig. 2 demonstrates that motor function was not impacted by the intrathecal injection procedure. Compared to the naïve rats, the general exploratory behaviors of rats with intrathecal implantation, such as the total activity (1874 ± 146), distance traveled (668 ± 29), and length of active time (1675 ± 42) in consecutive 45-minute intervals (n = 13) were not significantly different. In addition, no significant decreases were found in other parameters. On the average, rearing events and rearing times of 267 ± 16/45 min and 451 ± 77/45 min were found in naïve rats; they were 221 ± 19/45 min and 424 ± 61/45 min, respectively, in implanted rats.

### Intradermal injection of capsaicin changes exploratory behavior of rats

After noxious stimulation by intradermal injection of capsaicin in the left hind paw of rats that were implanted intrathecally with a catheter, spontaneous exploratory activity was severely affected compared to the naïve and intrathecally implanted rats (Fig. 2). Total activity greatly decreased from 1874 ± 147 to 1022 ± 81, and distance traveled decreased significantly from 668 ± 29 to 495 ± 9 in consecutive 45-minute intervals (Fig. 2A and 2B). Active time decreased from 1675 ± 42 to 1401 ± 25, whereas resting time increased from 1025 ± 42 to 1299 ± 25 (Fig. 2C and 2D). Rearing activity was also significantly impacted by capsaicin injection. Rearing time decreased greatly from 424 ± 61 to 206 ± 6, and rearing events decreased from 221 ± 19 to 116 ± 13 (Fig. 2E and 2F).

### The effect of intrathecal administration of a general inhibitor of PP2A on exploratory behavior

**Figure 3:**
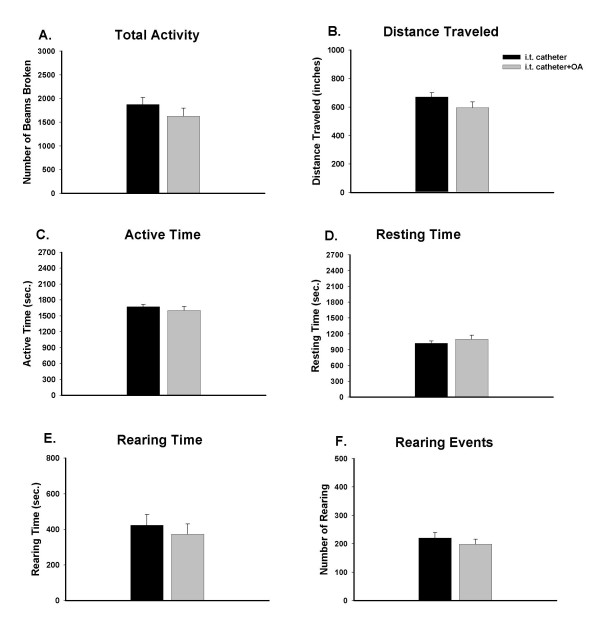
Effect of the PP2A inhibitor, okadaic acid (OA, 20 nM) on exploratory activity of rats during a consecutive period of 45 minutes (9 intervals, 5 min/interval). Compared to the intrathecal catheter implanted rats (n = 14), pretreatment with OA through an intrathecal catheter (n = 7) did not produce significant alterations in exploratory activity.

OA, a nonselective inhibitor of PP2A and PP1, was given intrathecally in a group of seven rats. Fig. 3 demonstrates that administration of OA had no effect on the spontaneous exploratory activity of the rats. None of the activity parameters in OA-administered rats, such as total activity (1628 ± 170), distance traveled (596 ± 40), active time (1603 ± 76), resting time (1097 ± 76), rearing time (373 ± 58), and rearing events (198 ± 18) in consecutive 45-minute intervals was significantly different when compared to the intrathecally implanted group.

### Decreased exploratory activity induced by capsaicin was potentiated by intrathecal administration of PP2A inhibitors

**Figure 4:**
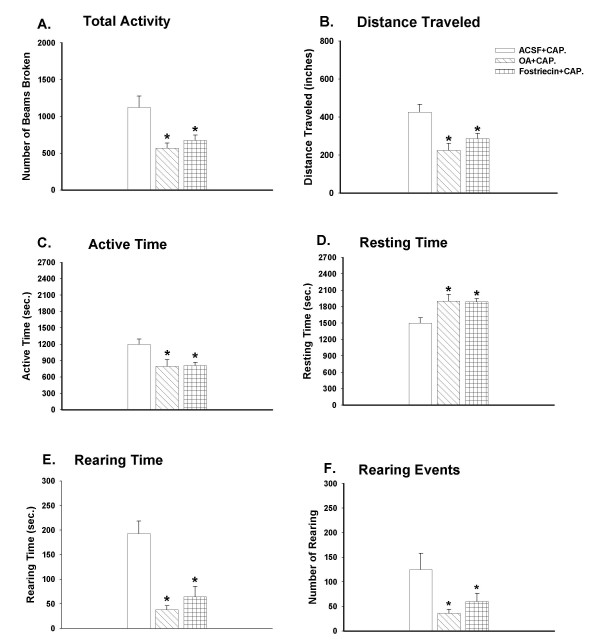
Effects of two PP2A inhibitors, OA and fostriecin, on the reduced exploratory activity of rats initiated by capsaicin injection during a consecutive period of 45 minutes (9 intervals, 5 min/interval). Compared to the rats in the ACSF-pretreated group (n = 7), the rats with either OA (20 nM, n = 7) or fostriecin (30 nM, n = 5) pretreatment displayed significantly decreased exploratory activity, including a decreased number of photobeams broken (A), shortened distance traveled (B), decreased active time (C), increased resting time (D), shortened rearing time (E), and decreased number of rearing events (F). *p < 0.05.

The bar graphs in Fig. 4 show the effects of PP2A inhibitors on the spontaneous exploratory activity of rats following capsaicin injection. Reduced exploratory behavior induced by capsaicin was aggravated by both the general inhibitor of PP1 and PP2A, OA, and the specific inhibitor of PP2A, fostriecin.

Three groups of rats were used in this experiment. Pretreatment with ACSF administered through the intrathecal catheter (n = 7) was done in the control group; OA (n = 9) or fostriecin (n = 5) was administered in the two experimental groups. Thirty minutes after the pretreatment with drugs infused through the intrathecal catheter, capsaicin was injected intradermally in the left hind paw of the rats. Animals were put into an activity chamber 30 min after the injection to monitor the movements.

Spontaneous exploratory activity in rats with OA pretreatment was greatly affected compared to the control group. Total activity significantly decreased from 1123 ± 160 to 570 ± 89; consequently the distance traveled decreased greatly from 426 ± 41 to 225 ± 37; active time decreased from 1200 ± 97 to 797 ± 123; and the resting time increased from 1500 ± 97 to 1903 ± 123 in consecutive 45-minute intervals. Rearing time in the OA group was also reduced greatly from 193 ± 26 to 39 ± 9, and the rearing events were reduced from 125 ± 33 to 36 ± 9. The measurements in the fostriecin-pretreated group were also significantly reduced compared to the control. For comparison, the total activity, distance traveled, active time, resting time, rearing time, and rearing events in fostriecin group were 674 ± 73, 286 ± 28, 900 ± 60, 1800 ± 60, 65 ± 21, 60 ± 18 respectively.

**Figure 5:**
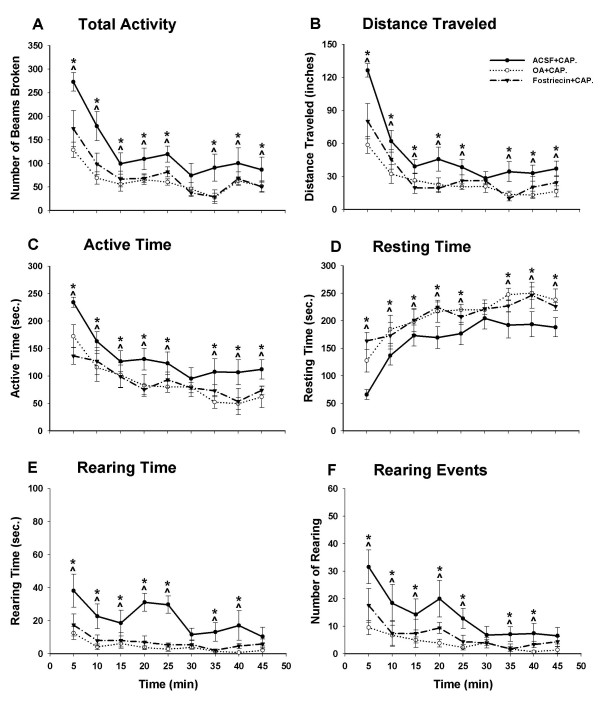
Effect of intrathecal infusion of the phosphatase inhibitors okadaic acid and fostriecin, or ACSF on decreased exploratory activity induced by capsaicin (0.1%, 50 μl) injection. Thirty min after capsaicin injection, okadaic acid (20 nM), fostriecin (30 nM), or ACSF was infused into the subarachnoid space of the spinal cord at the L5 segmental level of the rats. Compared to ACSF (n = 7), six parameters of spontaneous activity of the rats with okadaic acid (n = 7) and fostriecin (n = 5) infusion showed a significant decrease at every time interval. Each point represents the mean ± S.E.M. *p < 0.05.

The line graphs in Fig. 5 demonstrate the changes in exploratory behavior at each 5-min interval. When rats were placed in a novel environment, the spontaneous exploratory activity was high initially and slowly declined over the first 30 min. The activity in all groups of rats in the current study showed this same temporal pattern. The total entries, distance traveled, active time, rearing time, and rearing events were high in the first 20–25 min and then declined. In the groups treated with PP2A inhibitors, the exploratory activity was decreased compared with the ACSF-treated group. This reduction lasted throughout the 45-min block.

## Discussion

The results of this study showed that PP2A in the spinal cord plays an important role in the signal cascades that are activated following intradermal injection of capsaicin in the hind paw of rats. Based on the data presented, expression of PP2A in the spinal cord of rat starts to increase by 15 minutes after capsaicin injection and starts to return to baseline by about 1 hour.

With capsaicin injection, the spontaneous exploratory activity of rats was significantly reduced due to nociception. Following the injection, the number of rearing events and the rearing time were decreased. Measures of active time, total entries, and distance traveled were also decreased despite the fact that hind limb locomotor function was not impacted. In this model, secondary mechanical allodynia and hyperalgesia and primary thermal hyperalgesia would have occurred following capsaicin injection. So, during the process of central sensitization, a concurrent decrease in exploratory behavior would be expected, as seen in the current study. The reduction in exploratory activity seen in our model is in agreement with previous reports of exploratory behavior in other pain models. Palecek et al. [15] found a decrease in exploratory behavior following pain induced by colon inflammation and distention. Zhang et al. [16] showed reduced exploratory behavior in a model of pancreatitis. The decrease of movement with a normal ability to move results in less active time and more resting time, implying the development of pain following capsaicin injection. Intrathecal administration of PP2A inhibitors significantly aggravates pain, leading to a decrease in the total activity, traveled distance, active time, rearing time, rearing events, and an increase in the resting time of rats.

### PP2A expression in the spinal cord after noxious stimuli

Intradermal injection of capsaicin is a useful and reversible experimental model for the study of an inflammatory pain state that includes hyperalgesia and allodynia [17-20]. In the capsaicin model, central sensitization can be initiated by neural activity originating in the area of injection and carried by chemosensitive afferent fibers. The whole process relies on central pain mechanisms. It occurs after prolonged C-nociceptor input and depends on activation of wide dynamic range neurons in the dorsal horn of the spinal cord [21,22]. During central sensitization, a variety of signal transduction pathways in central neurons are activated, through which the excitability of central neurons is enhanced. It has been demonstrated that protein kinase C, NO/protein kinase G, protein kinase A, and calcium/calmodulin dependent kinase II (CaMKII) are all involved in this process. In addition, several types of ionotropic glutamate receptors become phosphorylated during central sensitization [20,23-31].

In this study, 15 minutes after capsaicin injection into the hind paw of rats, PP2A expression in the spinal cord is upregulated compared with the control group with a vehicle injection. In such a the short time period, the increased expression may not only reflect an increase of PP2A protein synthesis but also the posttranslational modification of PP2A subunits. The catalytic subunits of PP2A (PP2Ac) can be regulated mainly by three posttranslational modifications: methylation, phosphorylation of tyrosine and phosphorylation of threonine. By these modifications, PP2A activity and function can be regulated [32]. The antibody that we used in the Western Blot study recognizes the PP2A at 36 KDa, and it preferentially recognizes demethylated PP2A. This suggests that the increased PP2A expression may partly reflect an increased demethylation of PP2A.

The increased PP2A protein expression peaked at 30 min and it continued at least through 1 h following intradermal injection of capsaicin. This suggests that PP2A may also be involved in central sensitization after intradermal injection of capsaicin through modulation of the phosphorylation state of some critical proteins in the spinal cord.

### Capsaicin injection reduces exploratory activity of rats

In the present study, an automated photobeam activity system was used to test the rodent exploratory activity to evaluate behavioral changes induced by intradermal injection of capsaicin. The activity system was a useful tool for measurement of the exploratory activity for comparisons of control rats and rats with noxious stimulation of peripheral tissues, with or without pharmacological treatments. Capsaicin injection reduced the exploratory activity of rats by decreasing the total activity, traveled distance, active time, rearing time, and rearing events, while increasing the resting time in a 45-min period. Intradermal injection of capsaicin is a noxious stimulus that evokes not only ongoing pain (which lasts for about 15–20 minutes), but also a pain state in which there is increased sensitivity to mechanical stimuli. Intradermal injection of capsaicin can cause changes in behavioral responses of rats and increase responses of nociceptive projection neurons in dorsal horn of the spinal cord at a cellular level [15,17-20,22]. In our experiment, the exploratory activity of animals was tested 30 min after capsaicin injection. The changes of activity reflect the pain induced by mechanical stimulation of the paw during movement in the activity chamber instead of the pain evoked directly by the intradermal injection. In previous studies in our laboratory, intradermal injection of capsaicin did not cause any systemic effect, and the handling of rats during the injection had no effect [15]. On the other hand, the exploratory activity was not affected by the sham injection of vehicle (data not shown), which is consistent with the results of Palecek et al. [15]. The behavioral changes in exploratory activity are presumably due to nociceptive signals induced by capsaicin injection, and the changes reflect primary and/or secondary mechanical allodynia and hyperalgesia.

### PP2A inhibitors potentiate the reduction of exploratory activity that is induced by capsaicin injection

In the present study, PP2A inhibitors, either OA or fostriecin, did not themselves reduce the exploratory activity of the animals. On the other hand, pretreatment with PP2A inhibitors through an intrathecal catheter extended the duration of the decreases of the behavior induced by capsaicin. This suggests that without the peripheral noxious stimuli, the reactions that PP2A modulated would not initiate central sensitization directly, but they may be involved in the process of central sensitization by limiting the duration of this process.

It is well known that protein phosphatases function as key molecules in essential regulatory mechanisms in many cellular processes. Serine/threonine protein phosphatases remove phosphate groups from either serine or threonine sites following phosphorylation of target proteins and thus reverse protein phosphorylation [1,5,6,8,33,34]. Since protein dephosphorylation is an essential post-translational regulatory mechanism in signal transduction in the central nervous system that reverses the process of protein phosphorylation, protein phosphatases which mediate this process have gained more attention because their role in regulating protein phosphorylation is as important as that of protein kinases. PP2A is one of the most important protein phosphatases in the central nervous system, and it can regulate the activities of many kinases both *in vivo *and *in vitro*, such as ERK/MAPKs, PKA, PKC, IKB kinase, and Ca/calmodulin-dependent kinase [35-37]. Currently, glutamate receptors have become a main focus in pain research. The increases in responsiveness of dorsal horn neurons to mechanical stimulation of the skin after intradermal injection of capsaicin are due in part to the upregulation of phosphorylated glutamate receptors by a variety of protein kinases [38-41]. Our current findings of the effect of PP2A inhibitors on capsaicin-induced central sensitization suggest that PP2A has an effect on the duration of central sensitization. Although the mechanism is unclear, it may partially relate to the modulation of the phosphorylation of glutamate receptors, or regulation of the function of protein kinases by PP2A.

## Methods

### Experimental animals

Male Sprague-Dawley rats weighing 250–300 g were used in this study. The protocols for these experiments were approved by the Institutional Animal Care and Use Committee. These experimental protocols were also consistent with the ethical guidelines of the National Institutes of Health and the International Association for the Study of Pain. All animals used in the experiments were housed and maintained in accordance with the guidelines of the University of Texas Medical Branch (UTMB) Animal Care and Use Committee (ACUC).

### Intrathecal catheter implantation

The intrathecal catheters were made by joining three polyethylene tubes of different diameters and had a total length of 16 cm. The smallest diameter PE32 tubing (7.3–7.5 cm in length) (MICOR Inc., PA, USA) was inserted into the subarachnoid space of the spinal cord. The other end was connected with PE10 (3 cm) and then PE20 (8 cm) tubing (Becton Dickson, MD, USA) for a stepdown connection to a Hamilton syringe. Each joint of the catheter was sealed with epoxy glue. The catheter was dried, sterilized by immersion in 70% ethanol before insertion, and fully flushed with sterile saline prior to use. A length of stainless steel wire, whose diameter just fit into the PE32 tubing, was used during insertion.

Rats used in behavioral tests were anesthetized with sodium pentobarbital (50 mg/kg i.p.). The intrathecal catheter was inserted into the subarachnoid space of the spinal cord for the administration of drugs at the L5 segmental level according to the protocol of Yaksh et al. [42] as modified by LoPachin and Rudy [43]. To implant a catheter, the anesthetized rat was mounted in a stereotaxic instrument to hold the rat's head firmly. A midline incision was made beginning at a line joining the ears and extending about 2 cm in a caudal direction. The fascia was retracted from the skull. A superficial layer of neck muscles was exposed by the initial incision of the skin, and the muscles were separated by a midline incision beginning at the occipital crest and extending caudally about 2 cm. The separation of the superficial musculature exposed an underlying layer of muscles which could be easily separated along the midline by blunt dissection. Then, the muscles were freed from their point of origin on the occipital bone for about 0.5 cm on either side of the midline by using a scraping tool. While observing the back of the skull, the neck musculature was gently retracted and the atlanto-occipital membrane was exposed. In the midline, a small incision was made through the atlanto-occipital membrane, using the tip of a fresh 18- or 20-ga disposable needle as a cutting edge. Once the membrane was pierced, there was ample outflow of clear cerebrospinal fluid. The correct incision had to be carried out with considerable care to prevent cutting the dorsal surface of the medulla, which lies immediately beneath the membrane. The catheter was carefully advanced 7.3–7.5 cm into the spinal subarachnoid space from the incision. The tip of the catheter was placed at the T12-L1 vertebral level, which was estimated to be the L4/5 level of the spinal cord. When the catheter had been advanced 1–2 cm, a slight tension was applied to the tail of the animal to keep the animal's spine straight for the rest of the insertion. Once the catheter was inserted, the incision was sutured and the animal returned to the Animal Care Facility for 5 days to allow it to recover from the surgical procedure. The awake animals implanted with a catheter were checked for motor or sensory deficits after recovery. Animals with any sign of deficits were discarded.

### Administration of drugs

The drugs used in these experiments were serine/threonine protein phosphatase inhibitors: okadaic acid, a general inhibitor of PP2A and PP1 (20 nM, Calbiochem, dissolved in ACSF) and fostriecin, a potent PP2A inhibitor (30 nM, Calbiochem, dissolved in ACSF). Concentrations of these drugs were based on published dose response curves [44-46].

All drugs were administered through an intrathecal catheter implanted into the subarachnoid space of the spinal cord 30 minutes before injection of capsaicin. Drugs for intrathecal injection were dissolved in a 10 μl volume of ACSF containing the desired concentration of the agent. All drugs were injected over a 1–2 minute time period. A 10 μl injected volume has been shown to spread 2 cm rostral and caudally [42]. After intrathecal drug injection, the catheter was flushed with a subsequent 10 μl injection of ACSF. A Hamilton microinjection syringe (10 μl, Hamilton Co., Reno, NV) was used for all injections.

### Injection of capsaicin

Capsaicin (1%, 50 μl) was injected into the plantar surface of the glabrous skin of the left paw of halothane anesthetized rats [15]. Capsaicin was dissolved in Tween 80 (7%), alcohol (20%), and saline.

### Western blotting

Animals used in the Western immunoblotting study were deeply anesthetized with sodium pentobarbital (50 mg/kg, i.p.). The spinal cord was removed quickly at different timepoints after intradermal injection of capsaicin or vehicle. Laminectomy was done and the ipsilateral dorsal spinal cord tissue was placed in liquid nitrogen immediately and homogenized in 50 mM Tris buffer, pH 7.4 (0.1 mol/l EGTA, 0.14 μl/ml β-mercaptoethanol, 100 mol/l PMSF, and 0.2 mg/ml trypsin inhibitor). The homogenate was centrifuged at 13,000 g for 15 minutes, and the supernatant was decanted from the pellet and used for Western blot analysis. The concentration of protein in the homogenate was measured using a BCA kit (Pierce, Rockford, IL). Equal amounts of protein (60 μg) were size fractionated by 7.5% (w/v) sodium dodecyl sulfate-polyacrylamide gel electrophoresis (SDS-PAGE) and transferred onto a PVDF membrane (Bio-Rad, Hercules, CA). After blocking in the blocking buffer for 1 hour at room temperature, the blots are incubated with primary antibody for 1 hour at room temperature. The primary antibody to PP2A was monoclonal from Upstate (Charlottesville, VA) and primary antibody to β-actin was from Sigma (St. Louis, MO). Then the blots were washed three times for 30 minutes each in washing buffer and incubated with horseradish peroxidase conjugated IgG diluted in 2.5% (w/v) non-fat milk in washing buffer. The membranes were washed with buffer three times for 30 minutes and enhanced with a chemiluminescence reagent (ECL kit, Amersham, Arlington Heights, IL). Then the blots were exposed to autoradiographic film (Kodak, Rochester, NY), and the intensity of specific immunoreactive bands was quantified using densitometric scanning analysis. Densitometric units of specific bands were expressed relative to the values of background units. The relative density of the immunoblots from the spinal cord tissue of rats with injection of vehicle or capsaicin was calculated by dividing the density of the PP2A blots by the density of β-actin blots for the same conditions.

### Behavioral tests

The exploratory activity of the animals was recorded 5 days after intrathecal catheter implantation. For monitoring movements, two activity chambers (40 × 40 × 40 cm) were used and the data for motor activity were collected in the clear plastic chambers and analyzed using the software and hardware provided by the Photobeam Activity System (PAS) with Flexfield (San Diego Instruments, Inc.) coupled to a 486 Compaq computer. The PAS allows monitoring of the exploratory activity within the chamber by detecting the number of times photobeams are interrupted in an *x*, *y*, and *z *axis oriented grid system. There are 16 photobeams on each horizontal axis, which are arranged 4 cm above the chamber floor. Obstruction of these beams can be recorded as movements in the *x *and *y *planes. Movements along the *z *axis, which are called rearing events, can be detected by another set of beams located 12 cm above the chamber floor. When the animal remains still for 1 second or longer, this is defined as resting time. There are six different behavioral measures of exploratory activity to be recorded: the number of beams broken (counts), traveled distance, active time, resting time, rearing events, and rearing time. Since changes in one parameter are not reflected in every other parameter, the changes are evaluated for each parameter individually. The activity of each animal is recorded over nine consecutive 5-minute intervals for a total of 45 minutes. All the animals are tested at the same time during the day in a separate room where no people or other animals are present. The room is controlled at a stable temperature (70–72°F) and low level of noise. The animals are brought into the room just before the test, and the room is dark during the test. Between tests, the enclosure is cleaned with Cavicide and alcohol to eliminate urine and other olfactory cues from previous animals [15,16,47].

### Statistical analysis

The relative densities of Western blots from the tissue of the lumbar segments of rats at different time points (5 min, 15 min, 30 min, 60 min, and 90 min) were compared between vehicle and capsaicin groups. One-way ANOVA was performed followed by post hoc comparisons using Student-Newman-Keuls multiple comparisons test. All values are presented as mean ± S.E.M. A *p *value of less than 0.05 was considered significant.

All six activity measures were compared during the 45-min interval. Rats of the naïve group and intrathecal injection group were compared with Student's *t*-test. Student's t-test was also used to compare the responses of rats in the capsaicin group and the vehicle group. One-way ANOVA was performed to assess behavioral changes followed by post hoc comparisons using Student-Newman-Keuls multiple comparisons test. All treatment groups with capsaicin injection were compared to vehicle-treated rats. All data were expressed as mean ± S.E.M. A *p *value of less than 0.05 was considered significant.
